# Foramen of Huschke: It’s Relationship with Volume of Mastoid Pneumatization

**DOI:** 10.22038/IJORL.2023.66453.3282

**Published:** 2023-05

**Authors:** Şahinde Atlanoglu, Muhammed Fatih Topuz

**Affiliations:** 1 *Department of Radiology, Kutahya Health Sciences University, Kütahya, Turkey.*; 2 * Department of Otorhinolaryngology, Kutahya Health Sciences University, Kütahya, Turkey.*

**Keywords:** Computed tomography, Foramen of Huschke, Pneumatization of mastoid, Temporomandibular joint herniation into external auditory canal

## Abstract

**Introduction:**

The foramen of Huschke (FH) is a developmental bone defect located anteroinferior to the external auditory canal. In this study, the frequency of FH and the presence of TMJ herniation into the external auditory canal were investigated using high-resolution computed tomography (HRCT) of the temporal bone in patients with FH. In addition, it was aimed to determine whether there is a relationship between the degree of mastoid pneumatization and mastoid volume and the presence of FH.

**Materials and Methods:**

The HRCT images of 352 patients were retrospectively evaluated for the presence of FH and TMJ herniation into the external auditory canal. The degree of pneumatization was determined in 50 patients with FH and 53 patients without FH, and the mastoid volume was measured.

**Results:**

Of the 704 temporal bones, 50 (7.1%) were detected to have FH 16 (4.5%) on the right and 34 (9.7%) on the left. The incidence of FH was higher in women on the right than in men (p<0.01). There was strong correlation between the FH width and age for the left side (r=0.466, p<0.01). The mastoid volume was 3.2-15.9 cm3 in the patients with FH and 3.2-16.2 cm3 in those without FH. The degree of pneumatization and mastoid volume did not significantly differ between the both groups (p>0.05). TMJ herniation into the external auditory canal was detected in one of the patients with FH.

**Conclusions:**

We could not find a relationship between mastoid bone pneumatization and FH development. The presence of FH should be detected before TMJ and ear surgeries to prevent possible complications.

## Introduction

The tympanic bone develops through the process of membranous ossification. The tympanic membrane is formed in utero through the connection of the endoblastic and epiblastic sacs. During the first trimester of pregnancy, four ossification centers develop around the eardrum to form the U-shaped bone. At 35 weeks of gestation, this bone fuses with the squamous part of the temporal bone. The tympanic bone is incomplete at birth. Two protrusions appear anterior and posterior to the U-shaped bone and grow toward each other. Fusion occurs at one year of age at these two ossification points, which is the first step in the development of the external auditory canal (1). If the point of fusion does not correctly extend medially, an opening may occur at this point, which is referred to as the foramen of Huschke (FH) (2). FH usually closes completely before the age of five. Tympanic bone dehiscence at the fusion point should be considered an anatomical variant only after five years of age. The structure through which blood vessels or nerves pass is called foramen. FH is not a true foramen; bone defect or dehiscence is a more appropriate nomenclature (1). FH is located anteroinferior to the external auditory canal and posteromedial to the temporomandibular joint (TMJ) (3). FH is generally asymptomatic; however, if TMJ herniation develops from FH, otalgia and otorrhea may occur due to chewing (4,5). In a previous study involving 985 patients, complaints of pain, tinnitus, and ear fullness were reported in four female patients (0.4%) who had spontaneous TMJ herniation findings in the external auditory canal. Surgical reconstruction with titanium mesh was performed in these cases (6). TMJ synovial fluid may be the cause of otorrhea (7). In addition, it becomes easier for TMJ and parotid infections and tumors related to FH to spread to the external ear canal or for external ear infections and tumors to spread to the TMJ and parotid (8). The frequency of TMJ herniation in patients with FH has been reported to range from 2.9% to 27% (6,9). During TMJ arthroscopy, small diameter endoscopes can pass through the FH, causing tympanic membrane perforation and damage to the incus, malleus, and facial nerve. Therefore, it is crucial to determine the presence of FH before ear and TMJ surgery (3).

Pneumatization of the temporal bone, starting from the middle ear cavity and the region adjacent to the antrum, is completed around the age of 10 with the transition of air cells to the temporal bone. The degree of mastoid pneumatization varies from one person to another, which is explained by two theories: The first is Diamant’s theory of genetics, which states that badly mastoid pneumatization predisposes an individual to middle ear pathologies. The second, according to Wittmaac's environmental theory, the middle ear mucosa must be normal in order for the mastoid pneumotization process to progress. Middle ear pathologies lead to poor mastoid pneumotization (3,10). The development of tympanic bone and mastoid bone, which are in a close anatomical relationship, continues after birth. It is possible that these two bone segments affect each other (3).

This study aimed to investigate the frequency of FH and the presence of TMJ herniation into the external auditory canal using high-resolution computed tomography (HRCT) of the temporal bone in patients with FH, as well as to determine whether there is a relationship between the presence of FH and the degree of mastoid pneumatization and mastoid volume.

## Materials and Methods

**Fig 1:**
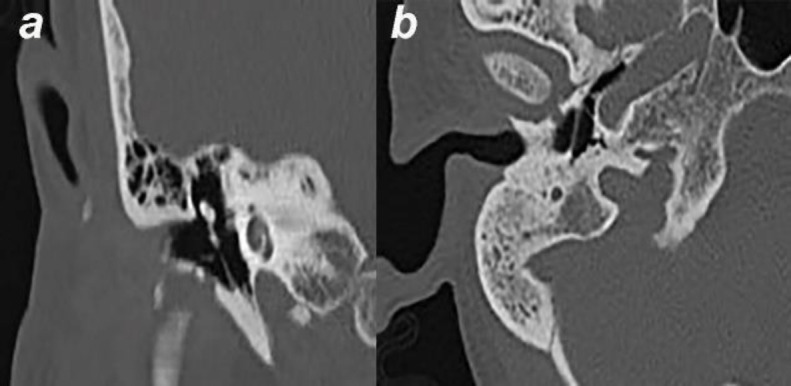
Computed tomography images showing the foramen of Huschke defect in the tympanic bone on the right side. (a) Coronal oblique reformatted image and (b) axial bone window

The images of 352 patients who underwent temporal bone HRCT between January 1, 2017 and December 31, 2019 were retrospectively evaluated by a 10-year-experienced radiologist. The images were obtained with a 16-slice multidetector computed tomography scanner (Aquilion, Toshiba Medical Systems, Otawara, Japan) using the following parameters: 120 kVp, 130 mAs, 220 field of view, 1 mm slice thickness. Scanning was performed parallel to the hard palate. The HRCT images were analyzed on a digital screen with 2048 x 2560 resolution. The temporal bone HRCT images were evaluated for the presence of FH and TMJ herniation into the external auditory canal. Since TMJ herniation into the external auditory canal was evident when the mouth was closed, attention was paid to keep the mouth closed. Images taken with the mouth open were excluded from the study. After detecting the presence of FH on axial images, its presence was confirmed on coronal oblique and sagittal oblique reformatted images (Figure 1a, b). 

In patients with FH, the width and side of FH were recorded, and those closer than 1 mm to the tympanic membrane were noted in the axial section. The medical histories of the patients with FH were obtained from the hospital database, and the presence of external ear canal, parotid and TMJ infections, and tumoral lesions was investigated. The images were reexamined one month apart regardless of the initial results. In addition to the presence of FH, the degree and volume of mastoid bone pneumatization were measured.

**Fig 2:**
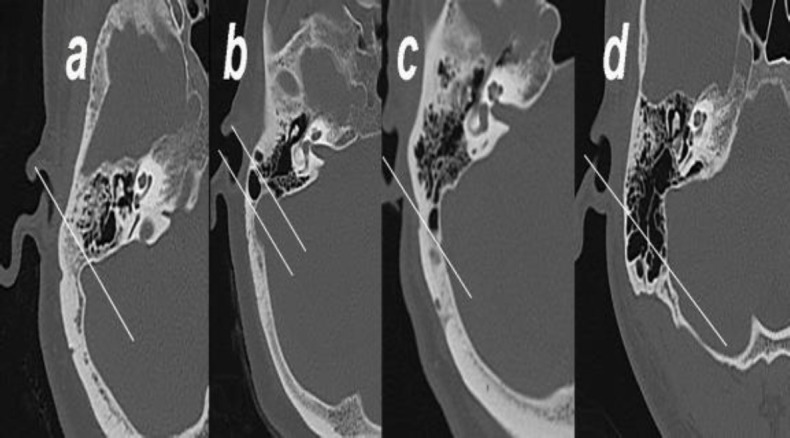
Axial bone window images showing the groups formed according to the pneumatization degree relative to the sigmoid sinus: (a) Group 1: Hypopneumatization, (b) Group: Moderate pneumatization, (c) Group 3: Good pneumatization, and (d) Group 4: Hyperpneumatization

The degree of mastoid bone pneumatization was classified according to the method described by Han et al. using the sigmoid sinus as a reference structure (10). According to this method, three lines extending 45 degrees anterolaterally from the most anterior, lateral, and posterior points of the sigmoid sinus were drawn in the section where the incudomalleolar joint was visualized as an ice cream cone shape on temporal tomography. The patients were divided into the following four groups according to the degree of pneumatization (Figure 2 a-d):

Group 1 (hypopneumatization): Pneumatization is located anteromedial to the most anterior line of the sigmoid sinus.

Group 2 (moderate pneumatization): Pneuma- tization extends to the lines passing the most anterior and lateral points of the sigmoid sinus.

Group 3 (good pneumatization): Pneumatization extends to the lines passing the most lateral and posterior points of the sigmoid sinus.

Group 4 (hyperpneumatization): Pneumatization extends beyond the sigmoid sinus postero- laterally.

**Fig 3:**
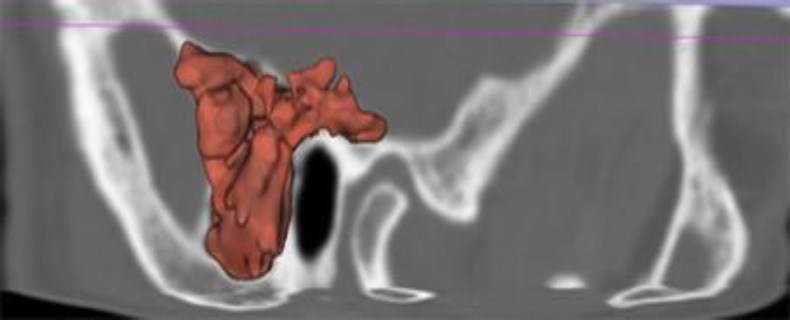
DICOM images transferred to the 3D SLICER-4.13.0 software to measure the mastoid cell volume. Manual segmentation was performed

To measure the mastoid cell volume, the Digital Imaging and Communications in Medicine (DICOM) images were transferred to 3D SLICER-4.13.0 software. For volume measurement, the lowest limit of the window level was determined as -1,000 HU and the upper limit as -200 HU. Manual segmentation was performed (Figure 3). 

The measurement of temporal bone pneumatization included the middle ear cavity, mastoid air cells, and petrous apex spaces, and the external ear canal was not included. The mastoid cell volume was automatically calculated by the software.

The gender and age of the patients included in the study were noted. Patients younger than 18 years and those who had a history of surgery disrupting the integrity of the tympanic bone were excluded from the study. 

*Statistical Analysis*

All data were analyzed with using “SPSS (Statistical Package for Social Sciences) for Windows 20.0 (SPSS Inc., Chicago, IL, USA), and p < 0.05 was accepted as the statistical significance limit. Mean ± standard deviation, minimum, and maximum values were used to represent descriptive continuous data, and numbers and percentages were used for discrete data. The Kolmogorov-Smirnov test was used to examine the conformity of the data with normal distribution. The t-test and the Mann-Whitney U test were used for group comparisons of continuous data. The chi-square and Fisher’s exact tests were conducted for group comparisons (cross-tables) of nominal variables. The Spearman correlation coefficient was used to examine the relationships between continuous data. The t-test was employed to compare the volume values in two groups, and one-way analysis of variance was performed to compare the groups according to the degree of pneumatization. The Tukey post-hoc test was conducted to determine the degree of pneumatization that caused significant differences. The paired-samples t-test was performed to compare the volume values between the right and left sides. The chi-square test was used for group comparisons (cross-tables) of nominal variables. The correlation between the degrees of pneumatization and the mastoid volume was analyzed using Spearman’s correlation coefficient. 

## Results

**Fig 4:**
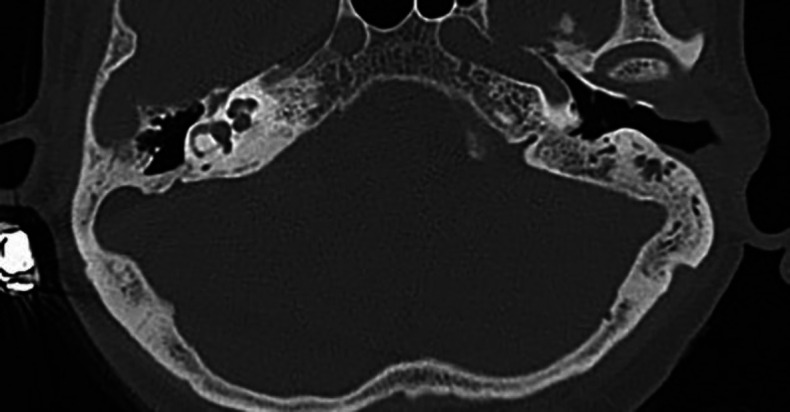
Temporomandibular joint herniation into the external auditory canal originating from the defect of the foramen of Huschke located on the left side

 A total of 352 patients (189 women and 163 men) were included in the study. The mean age was 49.67±13.82 years. FH was detected on the right side in 16 (4.5%) temporal bones and on the left in 34 (9.7%). Bilateral FH was detected in two patients. The mean FH width was 0.8 ±0.61 (0.21-2.06) mm on the right and 0.71±0.47 (0.27-2.21) mm on the left. The overall mean FH width of both sides was measured as 0.75±0.51 (0.21-2.21) mm. FH was closer than 1 mm to the tympanic membrane in only one patient. TMJ herniation into the external ear canal was observed in one patient with FH (2%) (Figure 4). 

When the medical histories of the patients were examined from the hospital system, no signs of external ear infection or TMJ infection were found. 

FH was found on the right side in 7.4% of female patients and 1.2% of male patients. There was a significant difference between women and men in terms of FH presence on the right side (p<0.01). Left FH was detected in 10.6% of women and 8.6% of men included in the sample, indicating no significant difference according to gender (p>0.05) (Table 1). There was no statistical difference in age between those with and without FH on both sides (Table 1).

**Table 1:** Distribution and comparison of the foramen of Huschke according to gender; comparison of age between the patients with and without the foramen of Huschke

	**Female**	**Male**	**p value**	**Age**	**p value**
	**n (%)**	**n (%)**		**Mean ± SD (min-max)**	
Foramen of Huschke on the right					0.133
Absent	175 (92.6)	161 (98.8)	0.006	49.43 ± 13.86 (18-80)	
Present	14 (7.4)	2 (1.2)		54.75 ± 12.42 (22-70)	
Foramen of Huschke on the left					0.182
Absent	169 (89.4)	149 (91.4)	0.528	49.35 ± 13.97 (18-80)	
Present	20 (10.6)	14 (8.6)		52.68 ± 12.16 (32-80)	
					

The mean left FH width was 0.74±0.55 mm among women and 0.66±0.33 mm among men. There was no significant difference between the left FH widths of the female and male patients (p>0.05). We were not able to compare the right FH width according to gender since it was only seen in two men. There was no statistically significant difference in age between the patients with and without FH on either side.

There was no correlation between the right FH width and age (r=0.199, p>0.05). However, a strong positive correlation was observed between the left FH width and age (r=0.466, p<0.01). The left FH width increased with increasing age.

For the mastoid volume measurement, 50 patients with FH and 53 without FH were included in the study. There was no significant difference between the patients with and without FH in relation to age and gender (p>0.05 for both).

In the FH group, no significant difference was found in the mastoid volume between the affected side and the contralateral side (p>0.903). The mastoid volume was 7.13±2.83 (3.8-13.4) cm^3^ on the affected side and 7.21±3.0 (3.2-15.9) cm^3^ on the contralateral side. There was also no significant difference between the volume values of the right and left sides among patients in the non-FH group (p>0.056). In this group, the mastoid volume was 7.66±2.85 (4.1-14.6) cm^3^ on the right and 8.41±3.84 (3.4-16.2) cm^3^ on the left. The FH and non-FH groups did not significantly differ in terms of the volume measurement (7.17±2.75 (3.2-15.9) cm^3^ and 8.00±3.41 (3.2-16.2) cm^3^, respectively; p>0.05). Pneumatization was not observed in 23.2% (n=22) of the patients in the FH group and 29.5% (n=31) of those in the non-FH group. Table 2 presents the degrees of pneumatization in the groups with and without FH. There was no significant difference in the degree of pneumatization between the FH and non-FH groups (p>0.05).

The mastoid volume differed significantly among the groups with different degrees of pneumatization (1, 2, 3, and 4; p<0.001). When further examined with the Tukey post-hoc test, a significant difference was observed between all the degrees (p<0.001). A positive correlation was detected between the degree of pneumatization and the mastoid volume (r=0.941, p<0.001). The relationship between the degree of pneumatization and the mastoid volume is shown in Table 2.

**Table 2:** Distribution of the degrees of pneumatization according to the study groups; comparison of the mastoid volume according to the degree of pneumatization

	**FH group**	**Non-FH group**	**p value**	**Volume**		**p value**
	**n %**	**n %**		**Mean ± SD**	**Min-max**	
**Degree of pneumatization**						
1	17 (23.3)	21 (28.4)		4.35 ± 0.80	3.2-6.3	
2	28 (38.4)	17 (23)	0.125	6.32 ± 0.88	4.1-8.1	<0.001ª
3	17 (23.3)	16 (21.6)		8.39 ± 0.75	6.5-10.4	
4	11 (15.1)	20 (27)		12.54 ± 1.74	9.1-16.2	

## Discussion

In addition to cholesteatoma and trauma, which are the most common causes of external auditory canal wall defects, FH is another cause. A rare TMJ herniation is usually associated with an anterior wall defect of the external auditory canal (6). It is typical for a polypoid mass to appear with the mouth open and disappear when the mouth is closed (11).

In the current study, FH was detected in 50 patients (7.1%) in 704 temporal bones. Park et al. (6) examined 985 temporal bones and reported the FH rate as 2.3%. Lacout et al. (1) reported the frequency of FH as 4.6% in 130 ears. The frequency of FH was higher in the current study than in both previous studies. Ertuğrul et al. (3) reported FH in 95 (13.3%) of 714 patients, which was higher than our finding. Similarly, in the current study, the mean FH width was measured as 0.75±0.51 (0.21-2.21) mm, while Lacout et al. (1) reported a mean axial diameter of 4.2 mm and Park et al. (6) determined the mean FH width as 3.06 mm. FH dimensions were significantly lower in the current study than in these two studies. One explanation for the variability between the reported incidence and size of FH may be differences in the lower-size limits of persistent FH used in each study. 

Another possible reason may be the effect of racial and genetic factors. Considering the difference between races in the incidence of frontal sutures in adults, it has been suggested that it may similarly affect the incidence of FH. The frontal suture, a variant of the normal in the skull, develops from a membranous process of ossification, in the same way as the tympanic plate from which FH is formed. According to Hashimoto et al. (12), the incidence of frontal sutures in Germans is 1.6 times higher than Japanese, 2.0 times higher than St Petersburg Russians and 7.3 times higher than Africans. The results of this study show that the membranous ossification process varies between races; racial factors may affect the incidence of persistent FH.

In the current study, TMJ herniation was detected in one patient. Park et al. (6) reported TMJ herniation in 26% of patients with FH in 985 temporal bones and 0.4% among all patients. The lower rate of TMJ herniation in the current study can be explained by the markedly lower FH width of the patients than in the study by Park et al. (6). 

Ertugrul et al. (9) reported the frequency of temporomandibular joint herniation as 2.9% in patients with FH, which is similar to the current finding. Jo et al. (13) reported that herniation was more common in patients with large defects, but it could also be seen in patients with a small defect size. Other factors such as mechanical stress that occurs during chewing can also cause herniation. In symptomatic cases, the defect can be closed with the preauricular approach in cases where the defect is large and with the endoaural approach in cases where the defect is small. Tragal cartilage, polypropylene, and titanium mesh are some materials used to close the defect (6, 14-16).

In the current study, no correlation was found between the right FH width and age. However, there was a strong positive correlation between the left FH width and age**. **This is in agreement with the findings by Ertuğrul et al. (9) that the prevalence of FH significantly increases with age, that persistent FH is not only congenital but could also be acquired, and that factors increasing osteolysis with aging and repetitive mouth movements could cause anterior wall thinning over years. We consider that FH is a congenital defect, and small FH defects can increase in diameter due to aging.

In our study, the rate of FH on the right side was higher among the female patients. FH is also reported to be more common in women (9-13). The higher incidence of right FH in women than in men may be related to the differences in the development of bone structures between men and women (3).

We determined the mean mastoid volume as 7.17±2.75 (3.2-15.9) cm^3^ in the FH group and 8.00±3.41 (3.2-16.2) cm^3^ in the non-FH group. The mean mastoid cell volume was reported as 15.28±5.34 (6.16-30.81) cm^3 ^by Han et al. (10) and 7.9±2.3 (4.0–14.0) cm^3 ^by Koç et al. (17). The value we obtained was similar to the latter but lower than the former. The lower mastoid volume of our sample than that reported in the study by Han et al. (10) may be due to racial and genetic factors, as well as the technique used to perform this measurement.

 In the current study, no significant difference was found between patients with and without FH in terms of the degree of pneumatization. In contrast, Ertuğrul et al. (3) reported that the degree of mastoid pneumatization was higher in patients with FH than in patients without FH. However, the authors did not perform mastoid volume measurements. In our study, we also measured the mastoid volume in both FH and non-FH groups but did not detect any significant difference. Further studies are needed to reveal the relationship between FH and the mastoid volume.

The limitations of the study include the HRCT slice thickness being taken as 1 mm. It may have been better to use a smaller slice thickness to perform temporal bone computed tomography. In addition, a single radiologist evaluated all the images, and therefore, inter-observer agreement could not be investigated. Lastly, we detected TMJ herniation in only one of the patients included in the study. The frequency of herniation can be better revealed by studies with a larger study population.

## Conclusion

In the current study, we could not find a relationship between mastoid bone pneumatization and FH development, which may affect each other. We think that due to aging, small FHs increase in size due to the mechanical effect of autolysis and chewing. The presence of FH should be detected before TMJ and ear surgeries to prevent possible complications.

The authors declare no conflict of interest.

The authors declare that they did not receive any financial support for the study.
